# Adult neurogenesis 20 years later: physiological function vs. brain repair

**DOI:** 10.3389/fnins.2015.00071

**Published:** 2015-03-06

**Authors:** Paolo Peretto, Luca Bonfanti

**Affiliations:** ^1^Neuroscience Institute Cavalieri OttolenghiOrbassano, Italy; ^2^Life Sciences and Systems Biology, University of TurinTorino, Italy; ^3^Department of Veterinary Sciences, University of TurinTorino, Italy

**Keywords:** neurogenesis, brain repair, neural plasticity, neurodegenerative diseases, fundamental research, neural specification, neuronal integration, neural stem cells

Two decades of intense investigation in the field of adult neurogenesis (AN) provided us with a fully renewed vision of brain plasticity, involving stem/progenitor cells capable of generating new neurons and glial cells throughout life. We know for sure that new neurons produced within canonical stem cell niches do play a significant role in cognitive tasks (learning/memory) operated by specific neural systems (Lepousez et al., 2013; Aimone et al., 2014). The fact that neural stem/progenitor cells (NSC) produce new elements that can integrate within some regions of the mature brain, replacing lost neurons/glial cells or adding to pre-existent neural circuits, appears extremely fascinating in the perspective of regenerative therapeutic approaches. Since the burst of investigations in AN/NSC field in the nineties, many neurobiologists addressed their studies on brain plasticity in the hope of brain repair, often discussing their results in a translational context. Nevertheless, in spite of striking efforts to clarify mechanisms/factors regulating AN and its physiological function, the question whether it can be exploited for healing neurologic diseases remains unsolved. More recent findings revealed additional examples of “non-canonical” neurogenesis and gliogenesis in various regions of the mammalian central nervous system (CNS; reviewed in Bonfanti and Peretto, 2011). These discoveries also open new hopes for brain repair, since the occurrence of spontaneous neuro-gliogenesis within the parenchyma does represent an endogenous source of progenitor cells even outside the restricted environment of canonical neurogenic sites. Nevertheless, parenchymal cell genesis remains substantially obscure as to its functional meaning(s) and outcome(s), and not yet exploitable for brain repair. Such an impasse largely resides on evolutionary discrepancies: most vertebrates use AN for brain repair as a byproduct of evolution, in addition to its physiological functions; mammals have lost such capacity, mainly because of unfavorable environments for repair/regeneration in their mature CNS (Bonfanti, 2011). A scarce perception of these facts might have produced misconceptions among scientists, sometimes leading to attitudes of unconditional optimism.

This Editorial is part of a Frontiers' research topic (and related e-book), gathering 18 articles which were intended to explore the relationships between actual existence of NCSs in mammals (playing homeostatic roles in AN and responding to pathological conditions) and lack of effective reparative outcome in terms of regenerative neurology. The topic was conceived starting from a critical pragmatism but also with the strong conviction that a promising future for AN research field is laying ahead, in the still far, yet conceivable, perspective of developing new therapeutic strategies. In our opinion, such hope is justified by the undeniable fact that our vision of brain structure and function has been fully reshaped after the discovery of structural plasticity involving NSC activity and AN outcomes. Accordingly, new dynamic, previously unsuspected impacts on brain physiology and pathology are expectable from such knowledge.

At present, multiple issues are still open. One first, fundamental question concerns the intrinsic limits of AN: does AN physiological functions include a role in brain repair? Some publications exploring canonical stem cell niches, such as the olfactory system (Oboti and Peretto, 2014; Sakamoto et al., 2014) and hippocampus (Vadodaria and Jessberger, 2014), strongly suggest that birth, specification, migration, and integration of young neurons is fashioned for these specific brain circuits with the demand for a special form of plasticity in the adult brain. This picture sets limits to the use of adult NSCs (at least for cell replacement strategies), leaving a possibility for neuronal integration only within anatomically-restricted regions and/or during short critical periods (Lois and Kelsch, 2014; Obernier et al., 2014; Turnley et al., 2014; Yamaguchi and Mori, 2014).

On these bases, most papers deal with the main constraints hampering exploitation of AN for neural cell replacement in aging and neurodegenerative diseases, including the quiescence and regional specification of progenitors, the failure in migration and integration of the newly born neurons. Hence, a second question could be: are stem cells restricted in fate limiting repair of different neuronal types? The first gap is in our understanding of quiescence vs. activity of the progenitors, and what is needed to control their *in vivo* regional specification. Very little is known about cell cycle parameters of adult neural, or any other somatic, stem cell. Some aspects under investigation, such as the variation of cell cycle length in pathological vs. physiological conditions, in gliogenic vs. neurogenic precursors, or in gray matter vs. white matter, can be linked to differently long periods of quiescence followed by re-entry in the cell cycle (Bragado Alonso et al., 2014). Also, the belief that neuronal precursors had extensive developmental plasticity has been rediscussed. Research during the last 20 years has shown that, in most cases, the fate of neurons is strongly determined and that it rarely changes (Lois and Kelsch, 2014). Even within the same lineage, the stem and progenitor cells are strikingly heterogeneous including NSCs that are dormant or mitotically active. These differences in NSC populations and activity states, including their role in neurogenesis and regeneration, how the different stem cells respond to aging, and how differences in cell signaling might contribute to adult NSC heterogeneity, are discussed by Giachino and Taylor (2014). In parallel, NSCs in the subventricular zone cannot acquire cortical, striatal or hippocampal properties following transplantation, rather, under normal physiological conditions they are highly specialized and regionally specified in a cell-autonomous manner to produce specific types of neurons destined for unique circuits within particular brain regions (Obernier et al., 2014).

A third question is: can neuron migrate, integrate and properly survive to provide functional repair? An important obstacle for brain repair in the adult brain is the long distances that frequently separate endogenous germinal niches, or sites of transplantation of progenitor cells, from the sites where new neurons would be required. Migration through the adult brain is limited to very specific paths and to specific subtypes of neurons and glial cells (Lois and Kelsch, 2014; Obernier et al., 2014). Neural precursor cells can respond to neural damage by proliferating, migrating to the site of injury, and differentiating into neuronal or glial lineages. However, after a month or so, very few or no newborn neurons can be detected, suggesting that even though neuroblasts are generated, they generally fail to survive as mature neurons and contribute to the local circuitry. The article by Turnley et al. addresses this lack of survival and integration as one of the major bottlenecks that inhibits effective neuronal replacement. They analyze factors that enhance newborn neuron survival and integration under normal physiological conditions, including neurotransmitters, cytoskeletal rearrangements, neurotrophins, and other modulators of neural plasticity (see also Eiriz et al., 2014). Other crucial and unresolved questions were addressed by our Perspective article (Peretto and Bonfanti, 2014): comparative analyses have not yet elucidated to which extent brain regenerative capability is a byproduct of evolution and to which extent the knowledge of mechanisms in physiological plasticity can implement brain repair. Also, it remains obscure how mature tissue environment can determine the outcome of AN in neurogenic niches vs. parenchymal regions, in mammals vs. non mammalian species, and, among mammals, in humans. Hence, defining the degree of lineage plasticity of adult NSCs and the signals that can override their intrinsic programming has important implications for developing cell replacement strategies based on the mobilization of endogenous cells.

A fourth theme regards the caveats to be considered before brain repair can be achieved from AN. Prospective solutions can fall into two domains: those trying to solve the above mentioned pitfalls (linked to specification, migration, integration of progenitors and their progeny, thus aiming at cell replacement) and those involving approaches alternative to the classic view of a regenerative neurology (not aiming at cell replacement). To solve the former problems both studies on cell autonomous properties (e.g., by shaping the neuronal differentiation of endogenous progenitors or using reprogrammed/engineered cells; Broccoli et al., 2014; Lois and Kelsch, 2014; Obernier et al., 2014) and environmental permissiveness (e.g., by extending or re-opening critical periods; Yamaguchi and Mori, 2014) can be addressed. On the whole, adult-born neurons themselves may not be useful to directly repair the brain, but learning from AN and developing new technological tools should guide our attempts to use engineered stem cells to achieve this goal. The alternative approaches could be linked to various functions of AN exerted in a wider context of brain plasticity both in physiology and pathology, a concept that is well introduced by the articles of Vadodaria and Jessberger (2014) and Butti et al. (2014). The dual role of AN cellular plasticity vs. cellular replacement for brain repair (remote plasticity) is analyzed by Quadrato et al. (2014). The open question about the ultimate impact of AN in the whole brain function is an issue which gained high interest in the last few years. The classic view of regenerative medicine aimed at using NSCs for replacing lost elements seems very hard to be realized in the near future for the mammalian CNS. By contrast, other functions/properties of neural progenitors could be exploited in exploring alternative roads to “structural” brain repair. Several articles discuss this issue under different points of view. Vadoaria and Jessberger, and Oboti and Peretto, address the physiological role of hippocampal and olfactory bulb neurogenesis and how it potentially contributes to the activity of different brain circuits. Endogenous stem/progenitor cells can be exploited for enhancing cognition in the diseased brain (Bordey, 2014). Within this context also falls the so-called bystander effect(s), which is analyzed in detail by Butti et al. (2014).

Another issue which has been rapidly expanding during the last few years is the occurrence in the mature CNS parenchyma of a vast population of progenitor cells still capable of division, which mainly provide continuous gliogenesis. Two articles deal with adult gliogenesis from NG2 cells, analyzing how these widely distributed parenchymal progenitors can act in brain physiology and repair (Nishiyama et al., 2014), and whether their purported role in oligodendrocytic cell replacement could be only one among other unknown functions in brain plasticity and repair (Boda and Buffo, 2014).

A final point regards the attitude of scientists in discussing the possible (at present non-existent) AN translational outcomes in their publications. Two levels of discussion should be addressed, concerning the production and interpretation of results (Peretto and Bonfanti, 2014), introducing another unanswered question: do we need adjustments in the peer review process of AN manuscripts to deal with gaps in experimental plan and result interpretation? A general survey of the main difficulties and pitfalls encountered in the translation of AN basic research knowledge accumulated during the last two decades to effective therapies for neurological diseases is also discussed in Lois and Kelsch (2014). A further analysis of the negative impact that science communication to the public can have on the society has been done in the Opinion paper by Cattaneo and Bonfanti (2014).

Our general feeling, as well as our conclusion, is that a future for research in AN have to necessarily pass through more fundamental research aimed at further understanding of the molecular/cellular mechanisms and the evolutionary logic of such type of plasticity before therapeutic approaches can be figured out and realized. Of course, researchers' attitude in supporting the importance and value of basic research even in the absence of immediate translational outcomes is fundamental in creating in patients and research financing institutions (private and public) the awareness that complex issues such as brain plasticity and repair in mammals cannot be addressed simply as the search for a therapy.

## Conflict of interest statement

The authors declare that the research was conducted in the absence of any commercial or financial relationships that could be construed as a potential conflict of interest.
